# Response to Letter to the Editor: “Normocalcemic Hyperparathyroidism: Study of its Prevalence and Natural History”

**DOI:** 10.1210/clinem/dgaa278

**Published:** 2020-05-18

**Authors:** Marian Schini, Richard Eastell

**Affiliations:** Department of Oncology and Metabolism, University of Sheffield, Sheffield, UK

Dear Editor,

We thank Drs. Zaidan and Wang for their interest in our study. There may be some confusion over the threshold for vitamin D deficiency. We used the threshold of 50 nmol/L or 20 ng/mL as recommended in the UK (1), the Institute of Medicine in the United States (2), and by the Fourth International Workshop on Asymptomatic Primary Hyperparathyroidism (3). In our group, we found that 3 of 11 patients identified with normocalcemic hyperparathyroidism (NPHPT) had 25(OH)D levels above 75 nmol/L on the index date for our study.

Drs. Zaiden and Wang mentioned that there were 2 patients with consistently high parathyroid hormone (PTH), but actually there were 7 such patients. In the remaining 4 patients (S0757, S0911, S3021, S3812), 3 were receiving vitamin D supplements and 2 of them had 25(OH)D >75 nmol/L at the time of diagnosis.

We are aware of the recent study evaluating free 25(OH)D in patients with NPHPT (4). These are interesting data and merit further study. However, free 25(OH)D is not part of the routine clinical practice at the moment, in part because we do not have an established threshold value.

Among the 4 patients found in our study to have consistent normocalcemia throughout their follow-up, none was on calcium supplements at the time of the diagnosis. Two reported relatively low calcium intake in their diet (less than 500 mg per day). From the remaining patients with intermittent hypercalcemia, only 1 was receiving calcium supplements on the index date for our study.

We do believe that these patients have a mild form of primary hyperparathyroidism. Our understanding is that in the case of parathyroid apoplexy, persistently high PTH returns to normal (5), which is not a pattern seen in our patients.

## Additional Information

***Disclosure Summary.*** M.S. received funding for her fellowship from the Medical Research Council Centre of Excellence for Musculoskeletal Ageing and from Osteoporosis 2000 support group and grant funding from Roche diagnostics. R.E. receives consultancy funding from IDS, Roche Diagnostics, GSK Nutrition, FNIH, Mereo, Lilly, Sandoz, Nittobo, Abbvie, Samsung, Haoma Medica and grant funding from Nittobo, IDS, Roche, Amgen and Alexion.
